# Supplementary material for: Elucidation of Gut Microbiota-Associated Lipids Using LC-MS/MS and 16S rRNA Sequence Analyses
Source: iScience. 2020 Nov 23;23(12):101841. doi: 10.1016/j.isci.2020.101841 (PMC7721639; doi:10.1016/j.isci.2020.101841)
Supplement: Document S1. Transparent Methods and Figures S1–S5 [file mmc1.pdf]

iScience

CellPress OPEN ACCESS

Article

Elucidation of Gut Microbiota-Associated Lipids Using LC-MS/MS and 16S rRNA Sequence Analyses

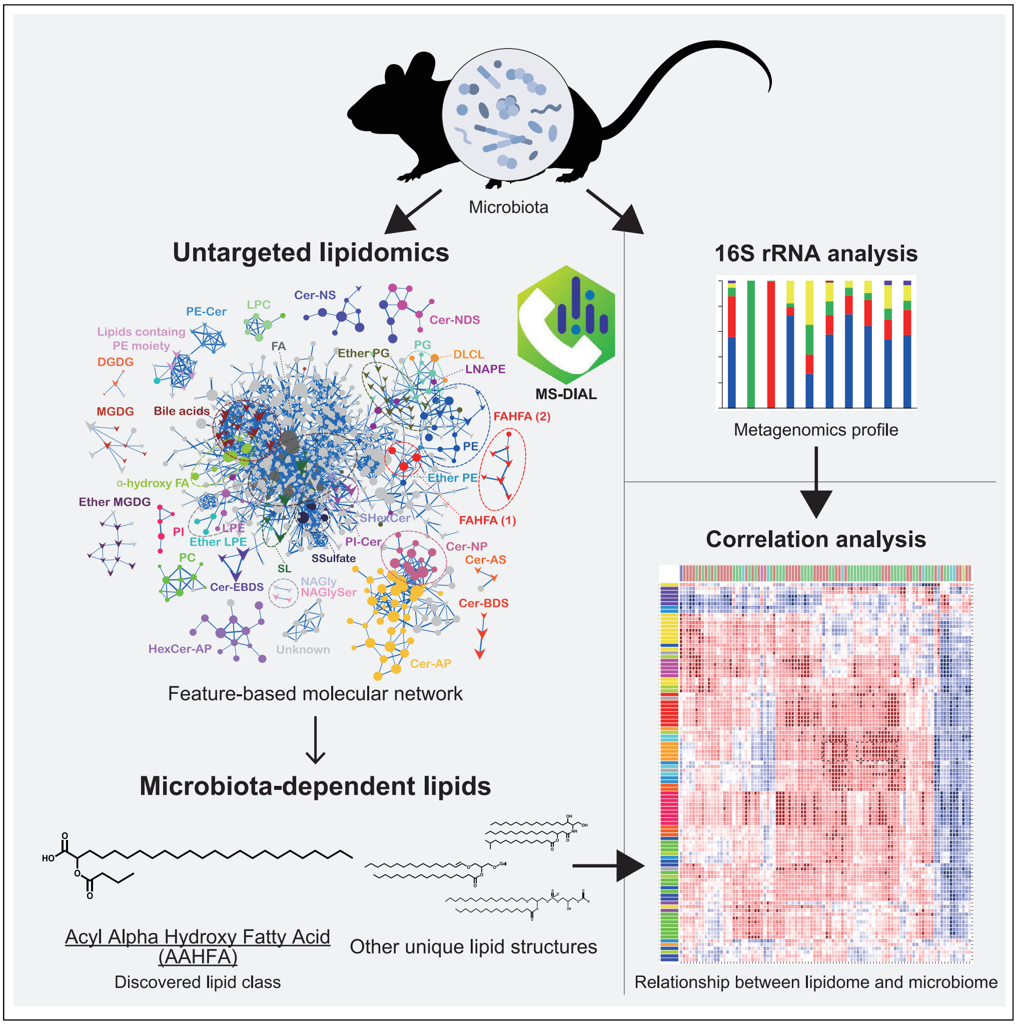

Shu Yasuda, Nobuyuki Okahashi, Hiroshi Tsugawa, ..., Hiroyuki Arai, Masahira Hattori, Makoto Arita

makoto.arita@riken.jp

HIGHLIGHTS

Feature-based molecular networking was explored in untargeted lipidomics analysis

Of all lipids, 24.8% of fecal lipids were decreased >10-fold in antibiotics-treated mice

Acyl alpha-hydroxy fatty acid (AAHFA) was identified as microbiota-specific lipid

With 16S rRNA data, we revealed the relevance of microbiome and lipidome in mice

Yasuda et al., iScience *23*, 101841
December 18, 2020 © 2020 The Author(s).
https://doi.org/10.1016/j.isci.2020.101841

Check for updates

iScience

CellPress
OPEN ACCESS

Article

Elucidation of Gut Microbiota-Associated Lipids Using LC-MS/MS and 16S rRNA Sequence Analyses

Shu Yasuda,[1,2,9] Nobuyuki Okahashi,[1,3,9] Hiroshi Tsugawa,[1,4] Yusuke Ogata,[1] Kazutaka Ikeda,[1,5] Wataru Suda,[1] Hiroyuki Arai,[2] Masahira Hattori,[1,6] and Makoto Arita[1,7,8,10,*]

SUMMARY

Host-microbiota interactions create a unique metabolic milieu that modulates intestinal environments. Integration of 16S ribosomal RNA (rRNA) sequences and mass spectrometry (MS)-based lipidomics has a great potential to reveal the relationship between bacterial composition and the complex metabolic network in the gut. In this study, we conducted untargeted lipidomics followed by a feature-based molecular MS/MS spectral networking to characterize gut bacteria-dependent lipid subclasses in mice. An estimated 24.8% of lipid molecules in feces were microbiota-dependent, as judged by > 10-fold decrease in antibiotic-treated mice. Among these, there was a series of unique and microbiota-related lipid structures, including acyl alpha-hydroxyl fatty acid (AAHFA) that was newly identified in this study. Based on the integrated analysis of 985 lipid profiles and 16S rRNA sequence data providing 2,494 operational taxonomic units, we could successfully predict the bacterial species responsible for the biosynthesis of these unique lipids, including AAHFA.

INTRODUCTION

The symbiotic bacteria in the gut affect host health and disease (Cho and Blaser, 2012; Honda and Littman, 2016). Genetic and environmental factors induce microbial imbalance called dysbiosis, which causes various diseases, including inflammatory bowel disease (IBD), diabetes, rheumatoid arthritis, and autism (Wang et al., 2011, 2015; Hsiao et al., 2013; Yoshimoto et al., 2013; Kim et al., 2014; Becattini et al., 2016; Pedersen et al., 2016; Rabot et al., 2016). Bacteria produce various bioactive metabolites, including short-chain fatty acids (SCFAs) and secondary bile acids (Chávez-Talavera et al., 2017; Levy et al., 2017; Sheng et al., 2017). These molecules are distributed to various host tissues via blood circulation and control host tissue homeostasis, disease phenotypes, and drug sensitivities (Kaddurah-Daouk et al., 2011; Fujisaka et al., 2018). In addition to hydrophilic metabolites, recent studies have revealed that bacteria also produce a series of hydrophobic lipids involved in host immune regulation, such as α-galactosylceramide (An et al., 2014), monoglucosyldiacylglycerol (Imai et al., 2018), and ceramide conjugated with phosphoinositol (PI-Cer) and phosphoethanolamine (PE-Cer) (Brown et al., 2019). Further, the structural specificity of these lipids has been indicated, as their different acyl chains are important determinants of their agonist activity on corresponding host receptors, as shown for SCFAs (Koh et al., 2016) and α-galactosylceramide (An et al., 2014). However, the comprehensive profiling of complex lipid structures in the gut environment and their biological relevance have not been fully addressed.

For a comprehensive understanding of the metabolic network in host-microbiota interactions, a deep metabolic profiling method is essential. Mass spectrometry (MS)-based untargeted analysis has been employed to detect unique bacterial metabolites (Wikoff et al., 2009; Dodd et al., 2017). Recent studies have provided the global metabolomic landscape of the gut microbiome in large cohort studies, revealing the dysregulated metabolism of bile acids and fatty acids in patients with IBD (Lloyd-Price et al., 2019; Franzosa et al., 2019). On the other hand, there are still many unknowns in MS data, and only 20% of mass ion signals are currently annotated, even with method blank subtraction and consideration of molecular adducts (Seitzer and Searle, 2019). Moreover, existing software programs and MS databases mostly cover host-derived metabolites but not much of the bacteria-derived ones. Thus, global metabolic profiling covering the

[1]RIKEN Center for Integrative Medical Sciences, Yokohama 230-0045, Japan

[2]Department of Health Chemistry, Graduate School of Pharmaceutical Sciences, University of Tokyo, 7-3-1, Hongo, Bunkyo-ku, Tokyo 113-0033, Japan

[3]Graduate School of Information Science and Technology, Osaka University, 1-5 Yamadaoka, Suita, Osaka 565-0871, Japan

[4]RIKEN Center for Sustainable Resource Science, Yokohama 230-0045, Japan

[5]Clinical Omics Unit, Kazusa DNA Research Institute, Kisarazu, Chiba, Japan

[6]Faculty of Science and Engineering, Waseda University, 3-4-1 Okubo, Shinjuku-ku, Tokyo 169-8555, Japan

[7]Division of Physiological Chemistry and Metabolism, Graduate School of Pharmaceutical Sciences, Keio University, Minato-ku, Tokyo 105-8512, Japan

[8]Graduate School of Medical Life Science, Yokohama City University, Tsurumi, Yokohama 230-0045, Japan

[9]These authors contributed equally

[10]Lead Contact

*Correspondence: makoto.arita@riken.jp

https://doi.org/10.1016/j.isci.2020.101841

© 2020 The Author(s).
This is an open access article under the CC BY license (http://creativecommons.org/licenses/by/4.0/).

molecular diversity of both host- and bacteria-derived metabolites is an emerging need for understanding metabolism in host-microbiota interactions.

Recently, we developed an advanced untargeted lipidomics platform that unbiasedly covers more than 8000 molecular species of 117 lipid subclasses (Tsugawa et al., 2020), although the relationship of lipids with the microbiome still remains a challenging issue. In this study, comprehensive profiling of lipids and the microbiome was achieved by liquid chromatography-tandem mass spectrometry (LC-MS/MS)-based untargeted lipidomics and 16S ribosomal RNA (rRNA) gene analyses. We employed feature-based molecular networking (FBMN, Nothias et al., 2020), which utilizes MS-DIAL feature detections followed by the MS/MS spectral networking algorithm (Tsugawa et al. 2019, 2020). The MS-DIAL-FBMN approach was used to (1) obtain the fecal lipidome landscape, including known and unknown lipids, (2) reveal structural features of known microbiota-derived lipids, and (3) elucidate unknown lipid structures associated with the gut microbiota. Furthermore, a correlation analysis of lipid profiles and 16S rRNA sequence data was performed to determine the bacterial species responsible for producing unique lipids present in the gut.

RESULTS

Characterization of Fecal Microbiome and Lipidome in Antibiotic-Treated Mice

In this study, the fecal microbiome and lipidome in antibiotic-treated mice were investigated using 16S rRNA sequencing and LC-MS/MS analyses. The administration of antibiotic-cocktails (Abx) containing ampicillin, vancomycin, neomycin, and metronidazole for two weeks substantially decreased the amount of bacterial 16S rRNA genes in feces (Figure 1A). Although 16S rRNA amplicon sequence analysis demonstrated the presence of Proteobacteria in the Abx-treated group (Figure 1B), most commensal bacteria were eliminated. The remaining Proteobacteria were confirmed as *Escherichia coli* (Data S1), suggesting an accidental outbreak of antibiotic-resistant *E. coli*, as reported in a previous study (Ayres et al., 2012). Single antibiotic treatment of either high or low dose showed differential effects on the fecal microbiome composition, while the total bacterial amount was kept within two-folds of control levels, except for the condition with high-dose of ampicillin (Figures 1A and 1B). At the phylum level, the relative abundances of Firmicutes and Bacteroidetes were decreased in the low dose ampicillin- or the high-dose vancomycin-treated groups, respectively (Figure 1B, Data S1). Principal coordinate analysis of 16S rRNA gene sequencing data also showed unique features of differential bacterial populations among the different antibiotic-treated groups (Figure S1). Score plots of Abx- and high-dose ampicillin- and vancomycin-treated groups were clearly separated from those of the other groups, with relatively mild perturbation of phylum composition in the PCo1 axis (Figure S1).

LC coupled to triple quadrupole (QqQ)- and quadrupole time-of-flight (QTOF)/MS platforms were used for targeted and untargeted LC-MS/MS analyses, respectively, to measure mouse fecal lipidome. The targeted analysis profiled a total of 136 molecules of free fatty acids and oxylipins, including HYA, a linoleic acid-derived bioactive metabolite produced by gut microbiota (Kishino et al., 2013). Of these, 23 fatty acid metabolites were >10-fold decreased in Abx treatment, with no increase observed in any metabolite (Table 1). Untargeted lipidomics using data-dependent acquisition provided a total of 10,010 chromatographic peak features after background subtraction, and a quarter of these was decreased in Abx treatment by > 10-fold (Table 1). Score plots of principal component analysis using the integrated lipid profiles from targeted and untargeted analyses showed that Abx and high-dose ampicillin and vancomycin groups were clearly distinguished from the other groups in the first principal component (PC1) axis. In addition, we found unique antibiotic treatment-dependent clusters in the three-dimensional score plots, suggesting that such treatments have substantial effects on metabolic profiles in addition to the perturbation of bacterial compositions (Figure 1C). Volcano plot between the control and Abx groups showed that 2,513 and 169 lipid ions were significantly decreased and increased over 10-folds, respectively (Figures 1D and Table 1). Thus, 26.7% of lipids in feces were estimated to be microbiota-dependent, as judged by > 10-fold increase or decrease in antibiotic-treated mice.

Bile acids, which are known to be metabolized by the microbiome, were identified based on retention times and MS/MS spectra matched with authentic standards. The peak abundances of unconjugated primary and secondary bile acids were significantly decreased in the Abx-treated group (ID 1–7 of Figure 1D). On the other hand, the amount of taurine-conjugated muricholic acid (MCA) was increased in the Abx-treated mouse feces (ID 8 of Figure 1D). The amount of taurine-conjugated cholic acid (CA) was also increased, although the fold change was less than 10-folds (ID 9 of Figure 1D). These results indicated a harmonized

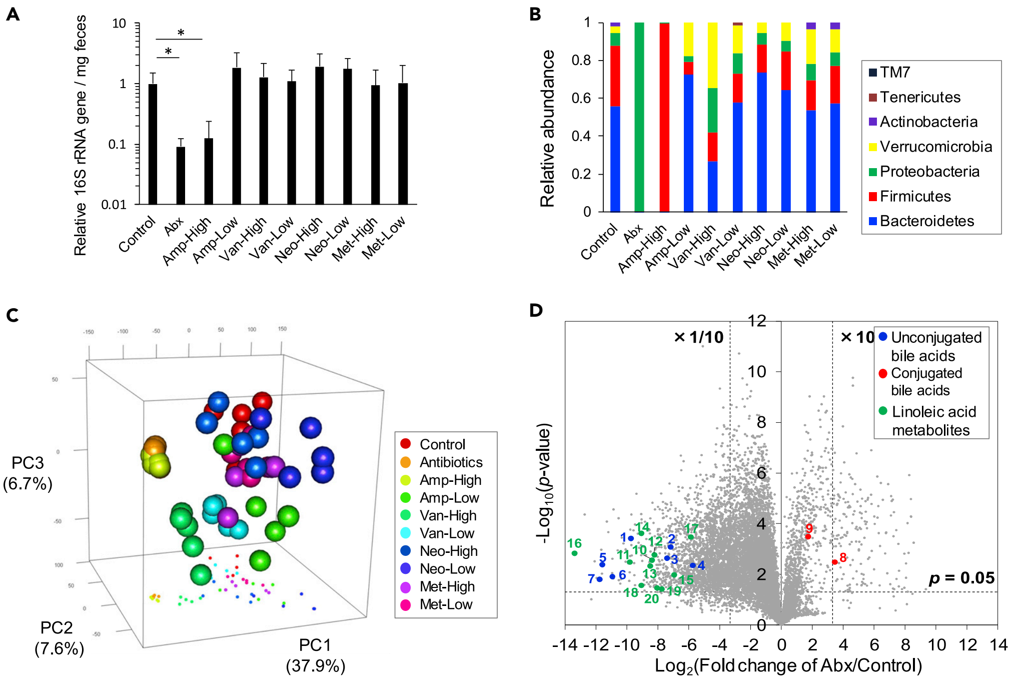

Figure 1. Microbiome and Lipidome Profiles in Antibiotic-Treated Mouse Feces

(A) The relative amount of 16S rRNA gene in the feces of mice treated with either low or high dose of ampicillin (Amp, 0.1 or 1 mg/mL), vancomycin (Van, 0.05 or 0.5 mg/mL), neomycin (Neo, 1 or 4 mg/mL), metronidazole (Met, 0.1 or 1 mg/mL), or their high dose mixture (Abx). The asterisk indicates the significance of the *t*-test ($p < 0.001$). Data are mean $\pm$ SEM ($n = 5$, except for the high dose Amp condition ($n = 3$)).

(B) The microbial composition in the feces of antibiotic-treated mice. Data are mean ($n = 5$).

(C) Principle component analysis of lipid profiles measured by targeted and untargeted lipidomics ($n = 5$ mice/condition).

(D) Volcano plot of lipids between Abx-treated and control mouse feces. Blue dots: unconjugated bile acids (1: MCA, 2: CA, 3: hyodeoxycholic acid (HDCA), 4: deoxycholic acid (DCA), 5: lithocholic acid (LCA), 6: isoLCA, 7: isoalloLCA). Red dots: conjugated bile acids (8: tauroMCA, 9: tauroCA). Green dots: linoleic acid metabolites (10: 10-hydroxy-*cis*-12-octadecenoic acid (HYA), 11: 10-hydroxyoctadecanoic acid (HYB), 12: 10-hydroxy-*trans*-11-octadecenoic acid (HYC), 13: 10-oxo-*cis*-12-octadecenoic acid (KetoA), 14: 10-oxo-octadecanoic acid (KetoB), 15: 10-oxo-*trans*-11-octadecenoic acid (KetoC), 16: 13-hydroxy-9-octadecenoic acid, 17: 13-oxo-9-octadecenoic acid, 18: 10,13-hydroxyoctadecanoic acid, 19: 10-hydroxy-*cis*-12-*cis*-15-octadecadienoic acid (αHYA), 20: 10-hydroxy-*trans*-11-*cis*-15-octadecadienoic acid (αHYC)). See also Figure S1 and Data S1.

bioreaction of the intestinal microbiome, where the deconjugation of primary bile acids followed by the conversion to secondary bile acids was coordinately catalyzed (Wahlström et al., 2016). Similarly, a series of unique linoleic acid metabolites, including HYA, were significantly reduced by Abx treatment (ID 10–20 of Figure 1D). These results demonstrated that a significant portion of fecal metabolites was dramatically reduced in Abx-treated mice.

Characterization of Microbiome-Derived Lipids via Molecular Spectrum Networking

The LC-QTOF/MS-based untargeted lipidomics data were processed by MS-DIAL (Tsugawa et al., 2020), which provided 5960 peak features with the MS/MS spectra information. Of these, we focused on 1671 and 152 peak features that were significantly decreased and increased by >10 fold after Abx treatment, respectively (Table 1). To determine the metabolic signatures of these lipids, including annotated and unknown molecules, we employed the FBMN technique (Figure 2). In FBMN, a node denotes a metabolic ion feature, and the nodes are linked if the MS/MS spectra have a high spectral similarity, indicating the existence of the same or similar substructure moieties.

By FBMN analysis, the MS/MS spectra of similar lipid subclasses were clustered by their fragmentation patterns, and unique metabolic profiles of each lipid subclass were revealed. Node shapes of triangle, reverse-triangle, and circle denote more than 10-fold increase, 10-fold decrease, and less than 10-fold change in

		The Number of Chromatographic Peak Features (Targeted + Untargeted Analyses)
Detected	Total	10,146 (136 + 10,010)
	MS2-acquired	6096 (136 + 5960)
	Identified	985 (136 + 849)
>10-folds decrease in Abx treatment ($p < 0.05$)	Total	2513 (23 + 2493)
	MS2-acquired	1692 (23 +1671)
	Identified	244 (23 + 225)
> 10-folds increase in Abx treatment ($p < 0.05$)	Total	169 (0 + 169)
	MS2-acquired	152 (0 + 152)
	Identified	1 (0 + 1)

Table 1. Summary of Lipidomics in this Study

the Abx-treated group, respectively, when compared to those in the control group. Lipid structures were characterized by curating the MS/MS spectra in the MS-DIAL software program (Tsugawa et al., 2020). The lipid nomenclature used in this study is available in Data S3 and at http://prime.psc.riken.jp/compms/msdial/lipidnomenclature.html. These results demonstrated that the abundances of bile acids, monoglucosyl/galactosyl diacylglycerol (MGDG), diglucosyl/galactosyl DG (DGDG), alkylacyl MGDG (Ether MGDG), alkylacyl phosphatidylglycerol (ether PG), sulfonolipid (SL, also known as sulfobacin), *N*-acyl glycine (NAGly) and *N*-acyl glycine serine (NAGlySer), PI-Cer, PE-Cer, acylated ceramide (AcylCer), ceramide alpha-hydroxy fatty acid-sphingosine (Cer-AS), ceramide beta-hydroxy fatty acid-sphinganine (Cer-BDS), and fatty acid esters of hydroxy fatty acid (FAHFA) were decreased by >10-fold in Abx-treated mouse feces (Figure 2). The structures of most lipid molecules were annotated by curating ESI(−)-MS/MS fragment ions in MS-DIAL (Tsugawa et al., 2020) to determine the lipid subclass and the *O*- and *N*-acyl chain properties of carbon number and ring/double bond equivalents (Data S2). As a result, we revealed a total of 226 lipid structures significantly perturbed by Abx treatment (Table 1). Although the structures have not been fully resolved, the lipid ion features containing the *m/z* 196.038 product ion in ESI(−)-MS/MS, indicating the existence of a PE-polar head moiety, were also decreased in the Abx-treated group.

Some of the drastically decreased lipid subclasses have been previously reported as bacterial lipids. For example, plasmalogen PG and MGDG are widely distributed in anaerobic bacteria, including intestinal *Clostridium* species (Řezanka et al., 2012). SL is known as a marker of *Alistipes* (Walker et al., 2017). The production of Cer-BDS, PE-Cer, and PI-Cer by *Bacteroides fragilis, B. thetaiotaomicron,* and *B. ovatus* has also been reported (Wieland Brown et al., 2013; An et al., 2014; Brown et al., 2019). In the MS/MS spectrum of AcylCer annotated by MS-DIAL, we found the characteristic fragment ion, revealing that the hydroxy moiety linked to the esterified acyl chain is located in the beta carbon position of the *N*-acyl chain; thus, the ceramide backbone can be termed Cer-BDS. Therefore, these AcylCer lipid subclasses in mouse feces were annotated as Cer-EBDS, where the character 'E' denotes the esterified moiety of fatty acid in Cer-BDS (Tsugawa et al., 2017, 2020). Ceramide clusters containing nonhydroxy-fatty acid-phytosphingosine (Cer-NP), ceramide containing nonhydroxy-fatty acid-sphinganine (Cer-NDS), ceramide containing nonhydroxy-fatty acid-sphingosine (Cer-NS), ceramide alpha-hydroxy fatty acid-phytosphingosine (Cer-AP), phosphatidylinositol (PI), dilysocardiolipin (DLCL), *N*-acyl lysophosphatidylethanolamine (LNAPE), and alpha-hydroxy fatty acids showed relatively mild changes under Abx treatment (>2-fold changes, Figure S3). Phosphatidylcholine (PC), lyso PC, lyso PE, and hexosyl Cer-AP did not change drastically (changes were less than 2-fold). Of interest, FAHFA molecules in two different clusters were observed in FBMN: one designated as "FAHFA (1)" displayed a mild change in abundance, while the other, annotated as "FAHFA (2)" showed a drastic decrease under Abx treatment (see the next section for further structure elucidation). These results demonstrated that the molecular networking approach based on untargeted lipidomics data is a powerful method not only for the characterization of lipid subclasses but also for the determination of bacteria dependency.

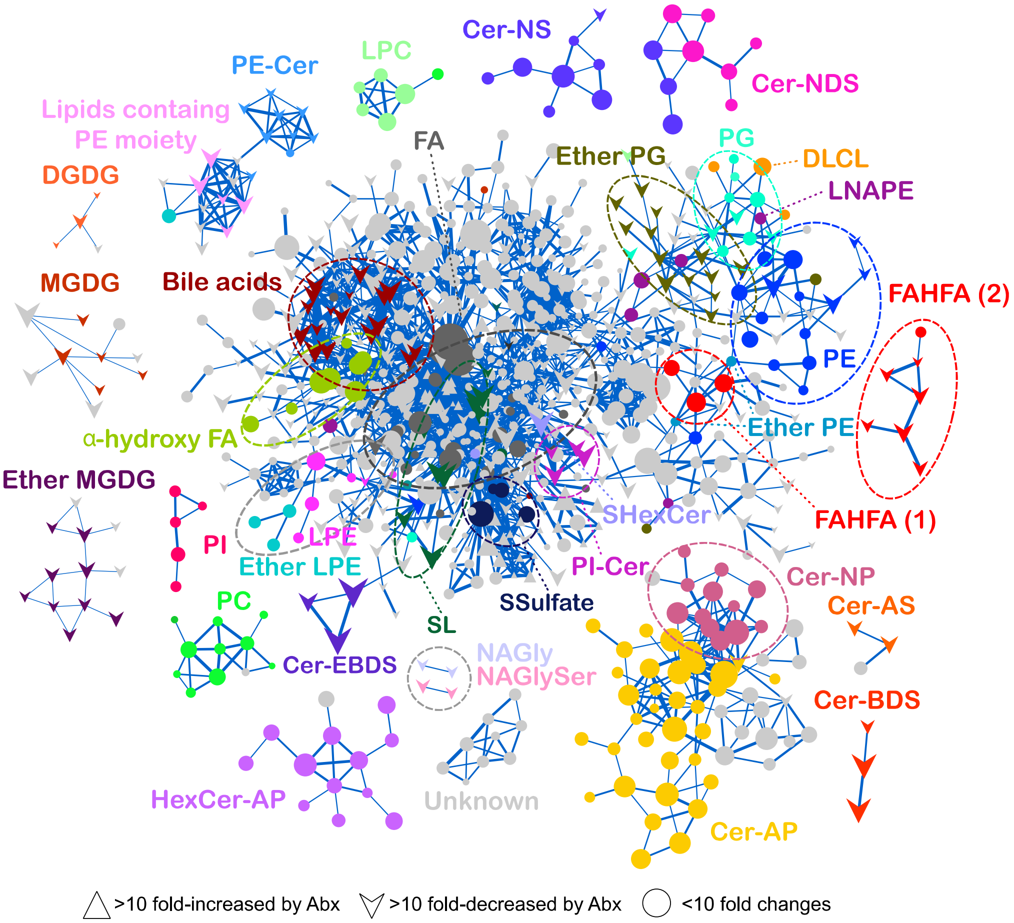

Figure 2. Feature-Based Molecular Spectrum Networking of Fecal Lipidome

Nodes corresponding to molecular species were linked based on the similarity of MS/MS spectra (Bonanza score > 0.85). The nodes of circle and up- and down-arrows represent lipid ions with less than 10-fold changes and more than 10-fold increase and decrease, respectively, in the Abx treatment group when compared to the control group. Node size and thickness of links denote the magnitude of measured ion intensity and Bonanza score, respectively. Nomenclatures of identified lipids are listed in Data S3. Mean intensity was used (n = 5). See also Figure S3 and Data S2.

Identification of a Discovered Lipid Subclass Acyl Alpha-Hydroxy Fatty Acid

The FBMN analysis revealed two distinct groups of FAHFA (Figure 2). However, only FAHFA (2) showed a higher microbiome-dependency, suggesting that the producers and structures of the two FAHFA groups were different. We observed the same product ion, indicating an esterified acyl chain moiety in both FAHFA (1) and FAHFA (2) MS/MS spectra (cleavage indicated in Figures 3A and 3B), while the chain length of the esterified acyl chain moiety was estimated as four (possibly butyrate) in FAHFA (2). Furthermore, we found a unique fragment ion (cleavage indicated in Figure 3B) in the FAHFA (2) MS/MS spectrum, which was estimated as the neutral loss of carboxylic acid moiety (46.008 Da). This fragmentation behavior enabled us to hypothesize that the hydroxy moiety of the fatty acid backbone of FAHFA (2) is located at the alpha carbon—the carboxylic acid neutral loss is not observed in well-known endogenous FAHFA molecules, such as palmitic-acid-9-hydroxy-stearic acid (9-PAHSA) and 5-PAHSA (Yore et al., 2014). To confirm the hypothesis, a compound with the proposed structure was chemically synthesized by the condensation of α-hydroxy fatty acid and butyric chloride (see Transparent Methods). Since the LC retention time and MS fragmentation pattern of the synthetic compound matched those of the endogenous signal annotated as FAHFA 4:0/24:0 in feces (Figure 3B), the hydroxy position of FAHFA (2) was proposed as the alpha carbon position. We also analyzed the synthetic butyric acid esters of β-hydroxy 24:0 and found that the retention time and fragmentation pattern were totally different from those of butyric acid esters of α-hydroxy 24:0 in the fecal samples (Figure S2). The lipid species grouped in

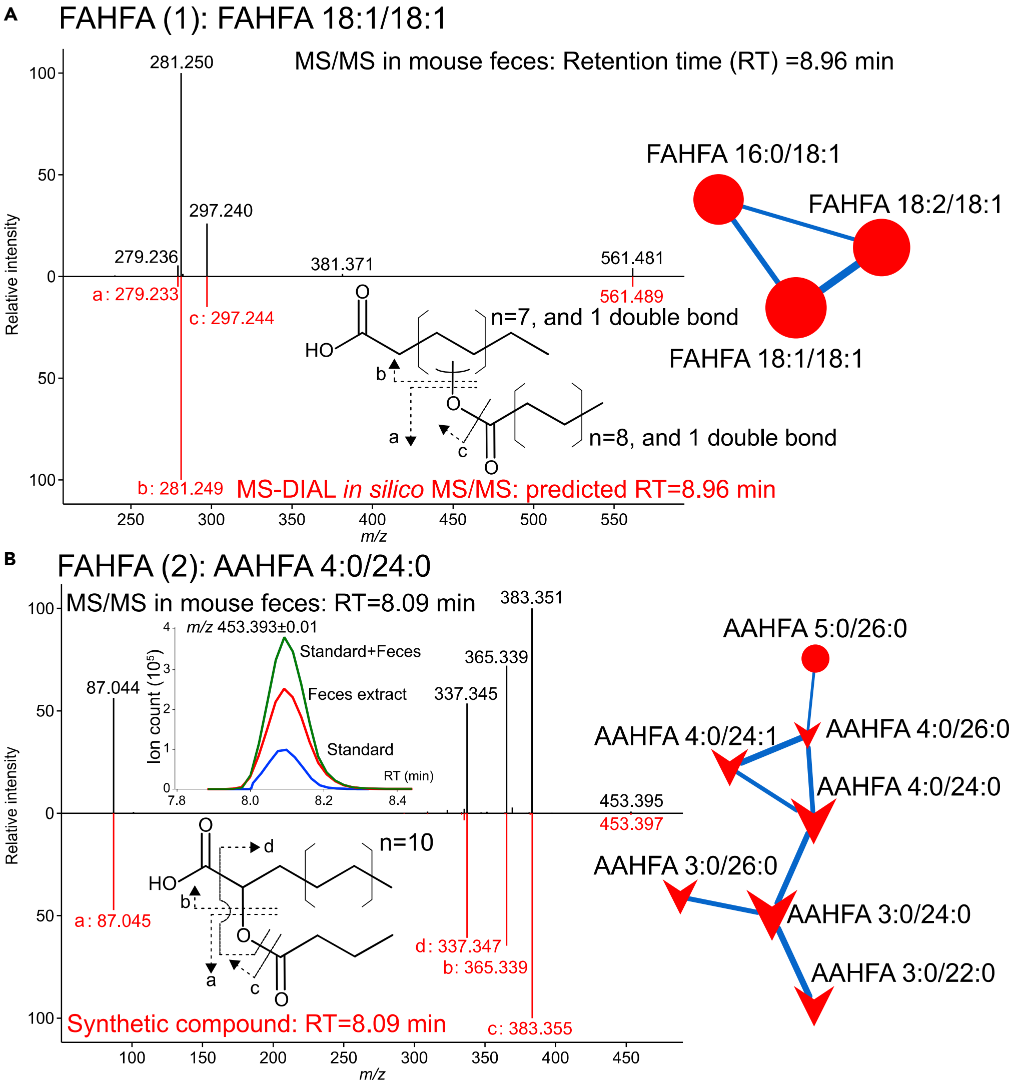

Figure 3. Identification of FAHFA and AAHFA Molecules in Feature-Based Molecular Network

(A) Measured (black) and *in silico* (red) MS/MS spectra of conventional FAHFA.

(B) Measured MS/MS spectra in the feces (black) and synthetic standard (red) of AAHFA. The chromatograms of the synthetic standard of AAHFA (blue), the fecal extract (red), and their mixture (green) are also described. The chemical structures represent the estimated fragmentation patterns corresponding to the measured mass spectra. The feature-based molecular networks of FAHFA and AAHFA presented on the right side were extracted from Figure 2.

FAHFA (2) included SCFA (C3-C5) in the esterified acyl chain and long carbon chain (C22-C26) in the α-hydroxy fatty acid backbone (Figure 3B). We also confirmed the existence of acetate esterified to α-hydroxy fatty acid (C24) in the lipidomics data, although it was not located in the same cluster of FAHFA (2) because the fragment ion of acetate (*m/z* = 59, FA 2:0) was not measured due to our mass range condition (*m/z* = 70–1750). Since the structure is not recorded in any metabolite database, including the human metabolome database, KEGG, and LIPIDMAPS, we referred to this microbiome-dependent new lipid subclass composed of SCFA and α-hydroxy fatty acids as *acyl alpha-hydroxy fatty acids*, abbreviated as AAHFAs. The levels of α-hydroxy fatty acids and their precursor, very long-chain fatty acids, were partially reduced in the feces of Abx-treated mice (15–40% of control, Figure S4), while the reduction rate was lower than that of AAHFA (<10% of control). The levels of SCFAs such as acetate, propionate, and butyrate were significantly decreased by 25.1-, 225- and 1790-folds, respectively (Figure S5), suggesting that the dramatic reduction in AAHFA levels in Abx-treated mice feces can be regarded as an additive effect of decreases in both α-hydroxy fatty acids and SCFAs.

CellPress
OPEN ACCESS

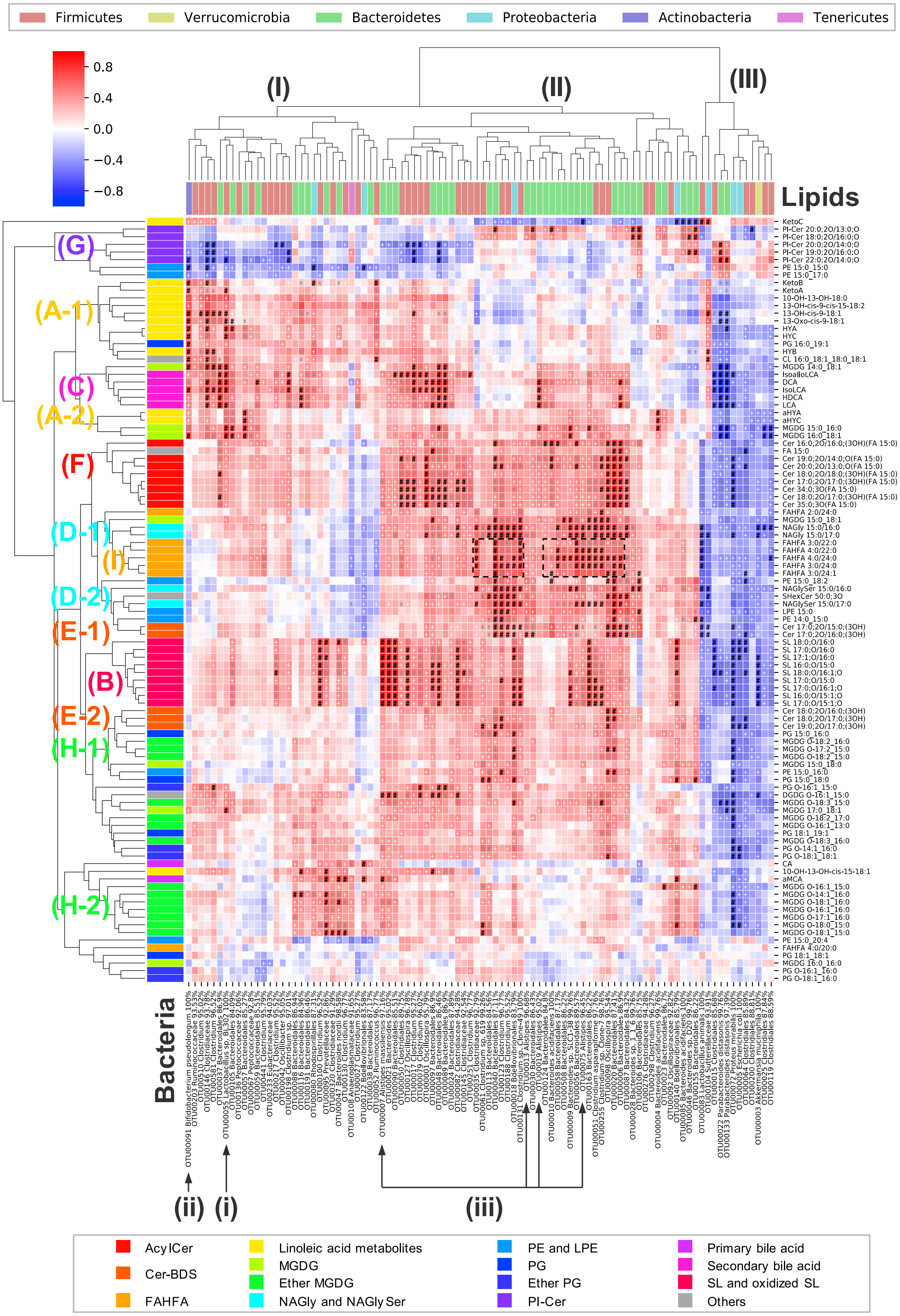

Figure 4. Correlation Analysis of Microbial Composition and Lipid Profiles

A heatmap representation of Spearman's rank correlation between the abundance of lipids and the read numbers of 16S rRNA gene sequence (n = 40). The bacteria operational taxonomic units (OTUs) of over 30 reads and the lipids with a >50-fold decrease in the Abx-treated group were used. The symbols indicate the significance (+: $p < 0.01$, *: $p < 0.001$, #: $p < 0.0001$). The annotation of bacteria was performed at the phylum, order, family, genus, or species level based on a sequence similarity threshold of 70%, 80%, 90%, 95%, or 97%, respectively. Vertical and horizontal color labels denote lipid subclasses and bacterial phyla, as described in the color legends.

Integrated Analysis of 16S rRNA Amplicon Sequences and Lipidomics Data

To investigate the relationship between lipidome and microbiome, the integrated analysis involving lipidomics and 16S rRNA amplicon sequencing was performed. The Spearman's rank correlation between microbial profiles and lipid profiles among the control and single antibiotic treatment groups was analyzed, where bacterial operational taxonomic units (OTUs) of over 30 reads and lipids with a >50-fold decrease in the Abx-treated group were mapped (Figure 4). The hierarchical clustering separated the bacterial profiles into three groups: (I) Firmicutes and Bacteroidetes, (II) Bacteroidetes-enriched, and (III) others. The molecular species of lipids were roughly classified according to the lipid categories (A-I of Figure 4). The amount of hydroxy- (HYA, HYB, and HYC) and oxo-forms (KetoA, KetoB, and KetoC) of linoleic acids (lipid class A-1 of Figure 4) positively correlates with the abundance of *Lactobacillus* sp. BL302 (bacteria i of Figure 4). Positive correlations were also found between α-linolenic acid metabolites (αHYA *etc.*, lipid class A-2) and *Lactobacillus* sp. BL302. The profile of *Bifidobacterium pseudolongum* (bacteria ii), which is a closely related species of *B. breve*, a known producer of HYB (O'Connell et al., 2013), also showed a positive correlation with linoleic acid metabolites (lipid class A-1). Similarly, a clear positive correlation was found between the abundance of SL (lipid class B) and its known producer *Alistipes* (bacteria iii) (Walker et al., 2017). The metabolic profiles of secondary bile acids containing DCA, HDCA, LCA, and LCA isomers (lipid class C) were positively correlated with the bacterial spectra of both the orders Bacteroidales and Clostridiales (bacteria group I and II). These results are consistent with those of a previous study that reported the involvement of *Bacteroides* and *Clostridium* bacteria in the deconjugation of conjugated bile acids (Masuda, 1981), which are subsequently converted to secondary bile acids. *N*-acyl amides, NAGly, and NAGlySer (lipid class D-1 and D-2), which are ligands for Toll-like receptor 2 present in the oral bacteria *Porphyromonas gingivalis* (Olsen and Nichols, 2018), were also enriched in Bacteroidales (bacteria group II), which highlights the ubiquitous presence of *N*-acyl amides in gut Bacteroidetes (Cohen et al., 2017). Correlation analysis also demonstrated a positive correlation between the abundance of various Bacteroidetes and sphingolipids containing odd-chain sphingoid base and/or fatty acids. For example, beta-hydroxy ceramide (Cer-BDS) and the esterified ceramide (Cer-EBDS) (lipid class E−1, and E−2, and F, respectively) showed positive correlations with Bacteroidales bacteria (bacteria group II). On the other hand, the bacterial correlation of PI-Cer (lipid class G) was different from that of Cer-BDS and Cer-EBDS, suggesting that the bacteria in different genera of the phylum Bacteroidetes produce different sphingolipid subclasses. The pattern of positive correlation was also different based on the chain length of PI-Cer molecules. Cer-BDS molecules (lipid classes E−1 and E−2) were classified into the different clusters based on the chain length of the sphingoid base- and *N*-acyl chain moieties. The total chain length of Cer-BDS in lipid class E−1, composed of C17 sphingoid base and C15–C16 *N*-acyl chain, was smaller than that in E−2, composed of C18–C19 sphingoid base and C16-C17 *N*-acyl chain. A similar feature was observed in ether MGDG with di- and mono-unsaturated fatty alcohols (lipid classes H-1 and H-2). This implies that the preferences of acyl chain length and degree of unsaturation of the metabolite precursor for the biosynthesis of even a single lipid subclass and the specificity would depend on the bacterial species. Finally, the abundance of the newly identified lipid AAHFA (lipid class I) was positively correlated with the bacteria in the Bacteroidales and Clostridiales orders (dashed square in Figure 4), indicating the relationship between these bacteria in AAHFA biosynthesis.

DISCUSSION

In this study, we revealed a global gut lipidome by LC-MS/MS-based untargeted lipidomics with molecular spectrum networking and characterized the relationship between bacterial composition and fecal lipid molecules by integrating 16S rRNA gene sequence data. Our untargeted lipidomics with the FBMN technique revealed 225 molecules with different lipid subclasses as microbiota-related lipids. The integrated analysis of lipidomics and 16S rRNA amplicon sequencing enabled us to predict the bacteria species responsible for the biosynthesis of the unique lipids. Moreover, our study identified a novel lipid subclass, namely AAHFA, derived from gut microbiota.

Molecular spectrum networking, where spectrum propagation is achieved to characterize unknowns, has increasingly been applied to plant metabolomics (Wang et al., 2016). Besides, we believe that the

CellPress
OPEN ACCESS

FBMN approach has a strong potential in untargeted lipidomics because the mass fragmentation pattern in the same lipid subclasses is similar among the various acyl chain properties (Tsugawa et al., 2020). Notably, the molecular spectrum networking revealed not only the microbiota-dependent lipid clusters but also discovered lipid structures, such as AAHFA, by their unique MS/MS fragmentation patterns.

The integrated analysis of lipidomics and 16S rRNA amplicon sequencing highlighted potential microbiota-derived lipid biosynthesis (Figure 4). Of note, many of our results, including the relevant HYA and *Lactobacillus*, SL and *Alistipes*, and ceramides and *Bacteroides*, were consistent with previous reports. Additionally, our study showed that the metabolic profile of Cer-BDS and Cer-EBDS in mouse feces was highly correlated with Bacteroidales bacteria (bacteria group II in Figure 4), while some Cer-EBDS molecules also had positive correlations with *Clostridium* bacteria (Figure 4). Based on these results, it could be speculated that the biosynthesis of Cer-EBDS using Cer-BDS as the substrate would take place in both Bacteroidales and *Clostridium* bacteria. Interestingly, there were lipid molecules of a single lipid subclass classified into two independent clusters based on acyl chain length and/or degree of unsaturation in the sphingoid base, *O*-acyl chain, and *N*-acyl chain moieties (Figure 4). Notably, the acyl chain-specific activity in bacterial lipids has also been reported, as described for SCFAs (Koh et al., 2016) and α-galactosylceramides (An et al., 2014). Therefore, it is important to determine precise lipid structures by MS-based lipidomics to understand host-microbiome interactions at the molecular level.

Our correlation analysis revealed a positive correlation of the newly identified lipid subclass AAHFA with the bacterial profiles of Bacteroidales and Clostridiaceae. This positive correlation was reasonable since the substrates of AAHFA, which are SCFAs such as butyrate and propionate, were reportedly produced by *Clostridium* and *Bacteroides* (Koh et al., 2016). The other part of AAHFA, namely α-hydroxy fatty acid, is potentially synthesized by bacterial CYP enzyme, which plays a role in oxygen detoxification in obligate anaerobic bacteria (Girhard et al., 2007). Moreover, α-hydroxy fatty acids can originate from the host-derived enzyme, fatty acid 2-hydroxylase (FA2H), which is highly expressed in the colon (Alderson et al., 2004). Our data indicated that the levels of α-hydroxy fatty acids were only partially reduced in Abx-treated mouse feces (Figure S4), suggesting that α-hydroxy fatty acids for AAHFA biosynthesis were derived from both bacteria and host. Furthermore, our results showed that the bacteria correlating with AAHFA metabolites were highly correlated with NAGly and NAGlySer molecules, which also have the structural backbone of fatty acid esters of hydroxy fatty acyls (lipid classes D-1 and D-2 in Figure 4). Importantly, NAGly molecules are biosynthesized in *Bacteroides* by *N*-acyltransferase (*glsB*), followed by the *O*-acyltransferase (*glsA*) enzymatic reaction (Lynch et al., 2019). Thus, it indicates that these bacteria would have an esterifying enzyme for α-hydroxy fatty acids to form AAHFAs. Although the activity of AAHFA was not determined in this study, structurally similar FAHFA is reported to be bioactive to stimulate insulin and GLP-1 secretion via the GPR120 receptor (Yore et al., 2014).

Consequently, we revealed a global fecal lipidome with the molecular spectrum networking and characterized the relationship between different gut microbiota and lipid profiles. The lipid molecular networking captured unique metabolic changes unbiasedly in the untargeted analysis and thus would be a powerful tool to open up a new avenue to discover potential links between microbial lipid metabolism and host biological phenotypes.

Limitations of the Study

The complete chemical assignment of lipid structures should be confirmed by chemically synthesized standards. The structures of *N*-acyl amides, ether MGDG, Cer-EBDS, and SL were characterized based on the exact mass and mass fragmentation patterns described in a previous study (Tsugawa et al., 2020). In addition, precise determination of acyl chain structures (straight, *iso*, or *anteiso*), unsaturation properties (unsaturated bond or cyclopropane), and sugar isomers (glucose or galactose, *etc.*) were not addressed in this study. Further, the precise determination of gut bacterial composition in mice was a challenge due to the lack of murine microbiome 16S rRNA reference (Xiao et al., 2015).

Resource Availability

Lead Contact

Further information should be directed to and will be fulfilled by the Lead Contact, Makoto Arita (makoto.arita@riken.jp).

Materials Availability

This study did not generate new unique materials.

Data Availability

MS data are available at the DropMet section of RIKEN PRIMe (http://prime.psc.riken.jp/) via the index of DM0032. 16S rRNA gene sequence data is available at DDBJ of the National Institute of Genetics (https://www.ddbj.nig.ac.jp/index-e.html) via the index of DRA010247.

METHODS

All methods can be found in the accompanying Transparent Methods supplemental file.

SUPPLEMENTAL INFORMATION

Supplemental Information can be found online at https://doi.org/10.1016/j.isci.2020.101841.

ACKNOWLEDGMENTS

This work was mainly supported by AMED LEAP under grant number JP18gm0010003 (for M.A.). The work was also supported partially by the JSPS Grant-in-Aid for Scientific Research on Innovative Areas "Lipo-Quality" (15H05897, 15H05898 for M.A.) and JSPS KAKENHI (18H02432, 18K19155 for H.T.). We thank our lab members, especially Akira Sen, Mie Honda, Aya Hori, Kanako Igarashi, Kaori Itaya, Yosuke Isobe, Masahiro Ueda, Kakou Ko, and Satoshi Morozumi, for their skillful technical supports and suggestions. We acknowledge Editage (www.editage.jp) for English language editing.

AUTHORS CONTRIBUTIONS

S.Y. and M.A. designed the study. S.Y. performed biological experiments. S.Y. and K.I. performed LC-MS/MS analyses, and Y.O., W.S., and M.H. performed 16S rRNA gene analyses. S.Y., N.O., and H.T. performed data analyses. S.Y., N.O., H.T., and M.A. wrote the manuscript. S.Y., N.O., H.T., H.A., and M.A. thoroughly discussed this project, and all authors helped improve the manuscript.

DECLARATION OF INTERESTS

The authors declare no competing interests.

Received: July 3, 2020
Revised: October 25, 2020
Accepted: November 18, 2020
Published: December 18, 2020

REFERENCES

Alderson, N.L., Rembiesa, B.M., Walla, M.D., Bielawska, A., Bielawski, J., and Hama, H. (2004). The human FA2H gene encodes a fatty acid 2-hydroxylase. J. Biol. Chem. *279*, 48562–48568.

An, D., Oh, S.F., Olszak, T., Neves, J.F., Avci, F.Y., Erturk-Hasdemir, D., Lu, X., Zeissig, S., Blumberg, R.S., and Kasper, D.L. (2014). Sphingolipids from a symbiotic microbe regulate homeostasis of host intestinal natural killer T cells. Cell *156*, 123–133.

Ayres, J.S., Trinidad, N.J., and Vance, R.E. (2012). Lethal inflammasome activation by a multidrug-resistant pathobiont upon antibiotic disruption of the microbiota. Nat. Med. *18*, 799–806.

Becattini, S., Taur, Y., and Pamer, E.G. (2016). Antibiotic-Induced changes in the intestinal microbiota and disease. Trends Mol. Med. *22*, 458–478.

Brown, E.M., Ke, X., Hitchcock, D., Jeanfavre, S., Avila-Pacheco, J., Nakata, T., Arthur, T.D., Fornelos, N., Heim, C., Franzosa, E.A., et al. (2019). Bacteroides-derived sphingolipids are critical for Maintaining intestinal homeostasis and symbiosis. Cell Host Microbe *25*, 668–680.e7.

Chávez-Talavera, O., Tailleux, A., Lefebvre, P., and Staels, B. (2017). Bile acid control of metabolism and inflammation in obesity, type 2 diabetes, dyslipidemia, and nonalcoholic fatty liver disease. Gastroenterology *152*, 1679–1694.e3.

Cho, I., and Blaser, M.J. (2012). The human microbiome: at the interface of health and disease. Nat. Rev. Genet. *13*, 260–270.

Cohen, L.J., Esterhazy, D., Kim, S.H., Lemetre, C., Aguilar, R.R., Gordon, E.A., Pickard, A.J., Cross, J.R., Emiliano, A.B., Han, S.M., et al. (2017). Commensal bacteria make GPCR ligands that mimic human signalling molecules. Nature *549*, 48–53.

Dodd, D., Spitzer, M.H., Van Treuren, W., Merrill, B.D., Hryckowian, A.J., Higginbottom, S.K., Le, A., Cowan, T.M., Nolan, G.P., Fischbach, M.A., et al. (2017). A gut bacterial pathway metabolizes aromatic amino acids into nine circulating metabolites. Nature *551*, 648–652.

Franzosa, E.A., Sirota-Madi, A., Avila-Pacheco, J., Fornelos, N., Haiser, H.J., Reinker, S., Vatanen, T., Hall, A.B., Mallick, H., McIver, L.J., et al. (2019). Gut microbiome structure and metabolic activity in inflammatory bowel disease. Nat. Microbiol. *4*, 293–305.

Fujisaka, S., Avila-Pacheco, J., Soto, M., Kostic, A., Dreyfuss, J.M., Pan, H., Ussar, S., Altindis, E., Li, N., Bry, L., et al. (2018). Diet, Genetics, and the gut microbiome drive dynamic changes in plasma metabolites. Cell Rep. *22*, 3072–3086.

Girhard, M., Schuster, S., Dietrich, M., Dürre, P., and Urlacher, V.B. (2007). Cytochrome P450 Monooxygenase from *Clostridium*

acetobutylicum: a new α-fatty acid hydroxylase. Biochem. Biophys. Res. Commun. *362*, 114–119.

Honda, K., and Littman, D.R. (2016). The microbiota in adaptive immune homeostasis and disease. Nature *535*, 75–84.

Hsiao, E.Y., McBride, S.W., Hsien, S., Sharon, G., Hyde, E.R., McCue, T., Codelli, J.A., Chow, J., Reisman, S.E., Petrosino, J.F., et al. (2013). Microbiota modulate behavioral and physiological abnormalities associated with neurodevelopmental disorders. Cell *155*, 1451–1463.

Imai, T., Matsumura, T., Mayer-Lambertz, S., Wells, C.A., Ishikawa, E., Butcher, S.K., Barnett, T.C., Walker, M.J., Imamura, A., Ishida, H., et al. (2018). Lipoteichoic acid anchor triggers Mincle to drive protective immunity against invasive group A Streptococcus infection. Proc. Natl. Acad. Sci. U S A *115*, E10662–E10671.

Kaddurah-Daouk, R., Baillie, R.A., Zhu, H., Zeng, Z.B., Wiest, M.M., Nguyen, U.T., Wojnoonski, K., Watkins, S.M., Trupp, M., and Krauss, R.M. (2011). Enteric microbiome metabolites correlate with response to simvastatin treatment. PLoS One *6*, e25482.

Kim, Y.G., Udayanga, K.G.S., Totsuka, N., Weinberg, J.B., Núñez, G., and Shibuya, A. (2014). Gut dysbiosis promotes M2 macrophage polarization and allergic airway inflammation via fungi-induced PGE2. Cell Host Microbe *15*, 95–102.

Kishino, S., Takeuchi, M., Park, S.B., Hirata, A., Kitamura, N., Kunisawa, J., Kiyono, H., Iwamoto, R., Isobe, Y., Arita, M., et al. (2013). Polyunsaturated fatty acid saturation by gut lactic acid bacteria affecting host lipid composition. Proc. Natl. Acad. Sci. U S A *110*, 17808–17813.

Koh, A., De Vadder, F., Kovatcheva-Datchary, P., and Bäckhed, F. (2016). From dietary fiber to host physiology: short-chain fatty acids as key bacterial metabolites. Cell *165*, 1332–1345.

Levy, M., Blacher, E., and Elinav, E. (2017). Microbiome, metabolites and host immunity. Curr. Opin. Microbiol. *35*, 8–15.

Lloyd-Price, J., Arze, C., Ananthakrishnan, A.N., Schirmer, M., Avila-Pacheco, J., Poon, T.W., Andrews, E., Ajami, N.J., Bonham, K.S., Brislawn, C.J., et al. (2019). Multi-omics of the gut microbial ecosystem in inflammatory bowel diseases. Nature *569*, 655–662.

Lynch, A., Tammireddy, S.R., Doherty, M.K., Whitfield, P.D., and Clarke, D.J. (2019). The Glycine lipids of Bacteroides thetaiotaomicron are important for fitness during growth in vivo and in vitro. Appl. Environ. Microbiol. *85*, 1–14.

Masuda, N. (1981). Deconjugation of bile salts by Bacteroides and Clostridium. Microbiol. Immunol. *25*, 1–11.

Olsen, I., and Nichols, F.C. (2018). Are sphingolipids and serine dipeptide lipids underestimated virulence factors of Porphyromonas gingivalis? Infect. Immun. *86*, 1–10.

Nothias, L.F., Petras, D., Schmid, R., Dührkop, K., Rainer, J., Sarvepalli, A., Protsyuk, I., Ernst, M., Tsugawa, H., Fleischauer, M., et al. (2020). Feature-based molecular networking in the GNPS analysis environment. Nat. Methods *17*, 905–908.

O'connell, K.J., Motherway, M.O.C., Hennessey, A.A., Brodhun, F., Ross, R.P., Feussner, I., Stanton, C., Fitzgerald, G.F., and Van Sinderen, D. (2013). Identification and characterization of an oleate hydratase-encoding gene from Bifidobacterium breve. Bioengineered *4*, 313–321.

Pedersen, H.K., Gudmundsdottir, V., Nielsen, H.B., Hyotylainen, T., Nielsen, T., Jensen, B.A.H., Forslund, K., Hildebrand, F., Prifti, E., Falony, G., et al. (2016). Human gut microbes impact host serum metabolome and insulin sensitivity. Nature *535*, 376–381.

Rabot, S., Membrez, M., Blancher, F., Berger, B., Moine, D., Krause, L., Bibiloni, R., Bruneau, A., Gérard, P., Siddharth, J., et al. (2016). High fat diet drives obesity regardless the composition of gut microbiota in mice. Sci. Rep. *6*, 1–11.

Seitzer, P.M., and Searle, B.C. (2019). Incorporating in-source fragment information improves metabolite identification accuracy in untargeted LC-MS data sets. J. Proteome Res. *18*, 791–796.

Sheng, L., Jena, P.K., Liu, H.X., Kalanetra, K.M., Gonzalez, F.J., French, S.W., Krishnan, V.V., Mills, D.A., and Wan, Y.J.Y. (2017). Gender differences in bile acids and microbiota in relationship with gender dissimilarity in steatosis induced by diet and FXR inactivation. Sci. Rep. *7*, 1–12.

Tsugawa, H., Ikeda, K., Tanaka, W., Senoo, Y., Arita, M., and Arita, M. (2017). Comprehensive identification of sphingolipid species by in silico retention time and tandem mass spectral library. J. Cheminform. *9*, 1–12.

Tsugawa, H., Nakabayashi, R., Mori, T., Yamada, Y., Takahashi, M., Rai, A., Sugiyama, R., Yamamoto, H., Nakaya, T., Yamazaki, M., et al. (2019). A cheminformatics approach to characterize metabolomes in stable-isotope-labeled organisms. Nat. Methods *16*, 295–298.

Tsugawa, H., Ikeda, K., Takahashi, M., Satoh, A., Mori, Y., Uchino, H., Okahashi, N., Yamada, Y., Tada, I., Bonini, P., et al. (2020). A lipidome atlas in MS-DIAL 4. Nat. Biotechnol. *38*, 1159–1163.

Wahlström, A., Sayin, S.I., Marschall, H.U., and Bäckhed, F. (2016). Intestinal crosstalk between bile acids and microbiota and its impact on host metabolism. Cell Metab. *24*, 41–50.

Walker, A., Pfitzner, B., Harir, M., Schaubeck, M., Calasan, J., Heinzmann, S.S., Turaev, D., Rattei, T., Endesfelder, D., Castell, W.Z., et al. (2017). Sulfonolipids as novel metabolite markers of Alistipes and Odoribacter affected by high-fat diets. Sci. Rep. *7*, 1–10.

Wang, Z., Klipfell, E., Bennett, B.J., Koeth, R., Levison, B.S., Dugar, B., Feldstein, A.E., Britt, E.B., Fu, X., Chung, Y.M., et al. (2011). Gut flora metabolism of phosphatidylcholine promotes cardiovascular disease. Nature *472*, 57–65.

Wang, Z., Roberts, A.B., Buffa, J.A., Levison, B.S., Zhu, W., Org, E., Gu, X., Huang, Y., Zamanian-Daryoush, M., Culley, M.K., et al. (2015). Non-lethal inhibition of gut microbial trimethylamine production for the treatment of atherosclerosis. Cell *163*, 1585–1595.

Wang, M., Carver, J.J., Phelan, V.V., Sanchez, L.M., Garg, N., Peng, Y., Nguyen, D.D., Watrous, J., Kapono, C.A., Luzzatto-Knaan, T., et al. (2016). Sharing and community curation of mass spectrometry data with global natural products social molecular networking. Nat. Biotechnol. *34*, 828–837.

Wieland Brown, L.C., Penaranda, C., Kashyap, P.C., Williams, B.B., Clardy, J., Kronenberg, M., Sonnenburg, J.L., Comstock, L.E., Bluestone, J.A., and Fischbach, M.A. (2013). Production of α-galactosylceramide by a prominent member of the human gut microbiota. PLoS Biol. *11*, e1001610.

Wikoff, W.R., Anfora, A.T., Liu, J., Schultz, P.G., Lesley, S.A., Peters, E.C., and Siuzdak, G. (2009). Metabolomics analysis reveals large effects of gut microflora on mammalian blood metabolites. Proc. Natl. Acad. Sci. U S A *106*, 3698–3703.

Xiao, L., Feng, Q., Liang, S., Sonne, S.B., Xia, Z., Qiu, X., Li, X., Long, H., Zhang, J., Zhang, D., et al. (2015). A catalog of the mouse gut metagenome. Nat. Biotechnol. *33*, 1103–1108.

Yore, M.M., Syed, I., Moraes-Vieira, P.M., Zhang, T., Herman, M.A., Homan, E.A., Patel, R.T., Lee, J., Chen, S., Peroni, O.D., et al. (2014). Discovery of a class of endogenous mammalian lipids with anti-diabetic and anti-inflammatory effects. Cell *159*, 318–332.

Yoshimoto, S., Loo, T.M., Atarashi, K., Kanda, H., Sato, S., Oyadomari, S., Iwakura, Y., Oshima, K., Morita, H., Hattori, M., et al. (2013). Obesity-induced gut microbial metabolite promotes liver cancer through senescence secretome. Nature *499*, 97–101.

Řezanka, T., Křesinová, Z., Kolouchová, I., and Sigler, K. (2012). Lipidomic analysis of bacterial plasmalogens. Folia Microbiol. *57*, 463–472.

iScience, Volume *23*

Supplemental Information

Elucidation of Gut Microbiota-Associated Lipids Using LC-MS/MS and 16S rRNA Sequence Analyses

Shu Yasuda, Nobuyuki Okahashi, Hiroshi Tsugawa, Yusuke Ogata, Kazutaka Ikeda, Wataru Suda, Hiroyuki Arai, Masahira Hattori, and Makoto Arita

Supplemental figures

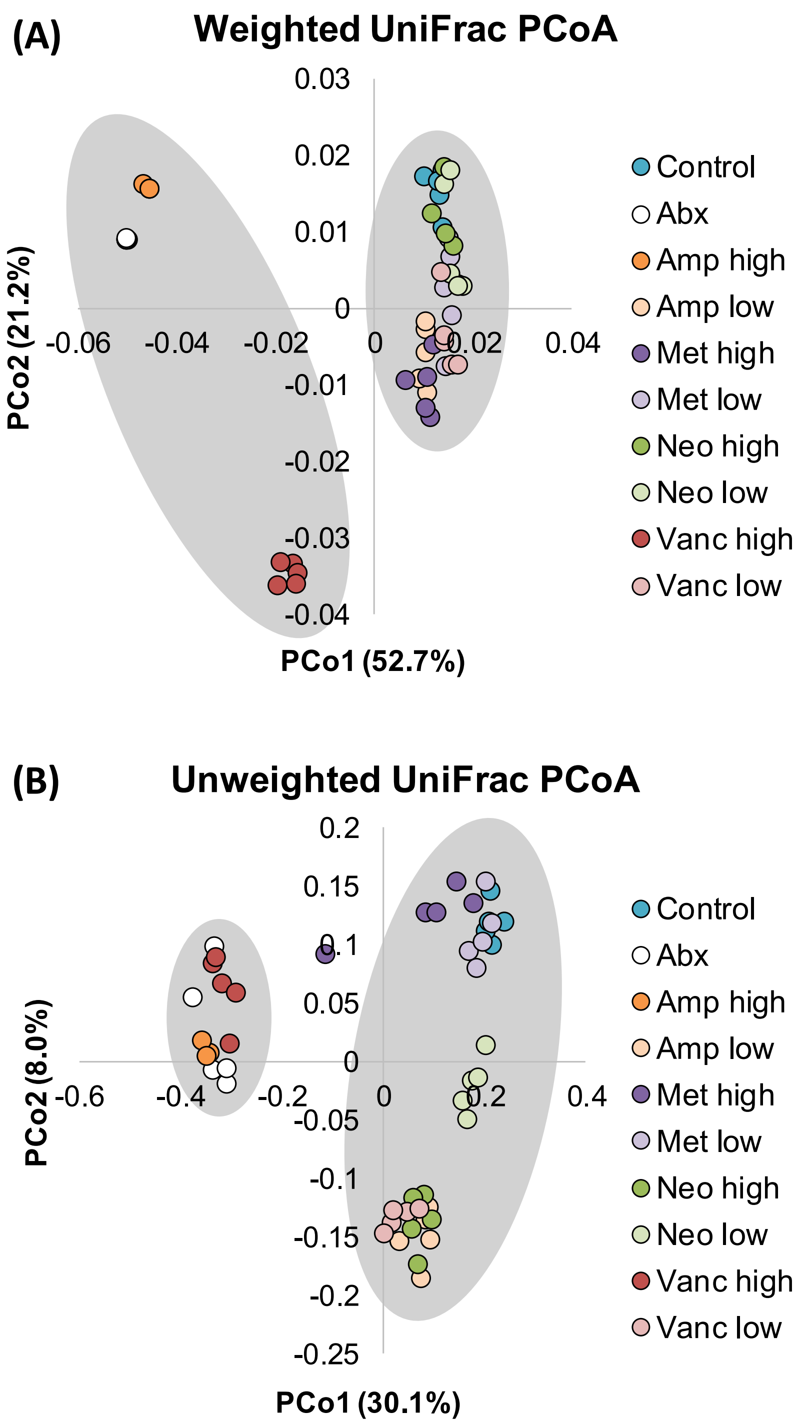

Figure S1. Principle coordinate analysis, Related to Figure 1

Score plots of principle coordinate analysis of **(A)** weighted and **(B)** unweighted UniFrac distance of 16S rRNA genes.

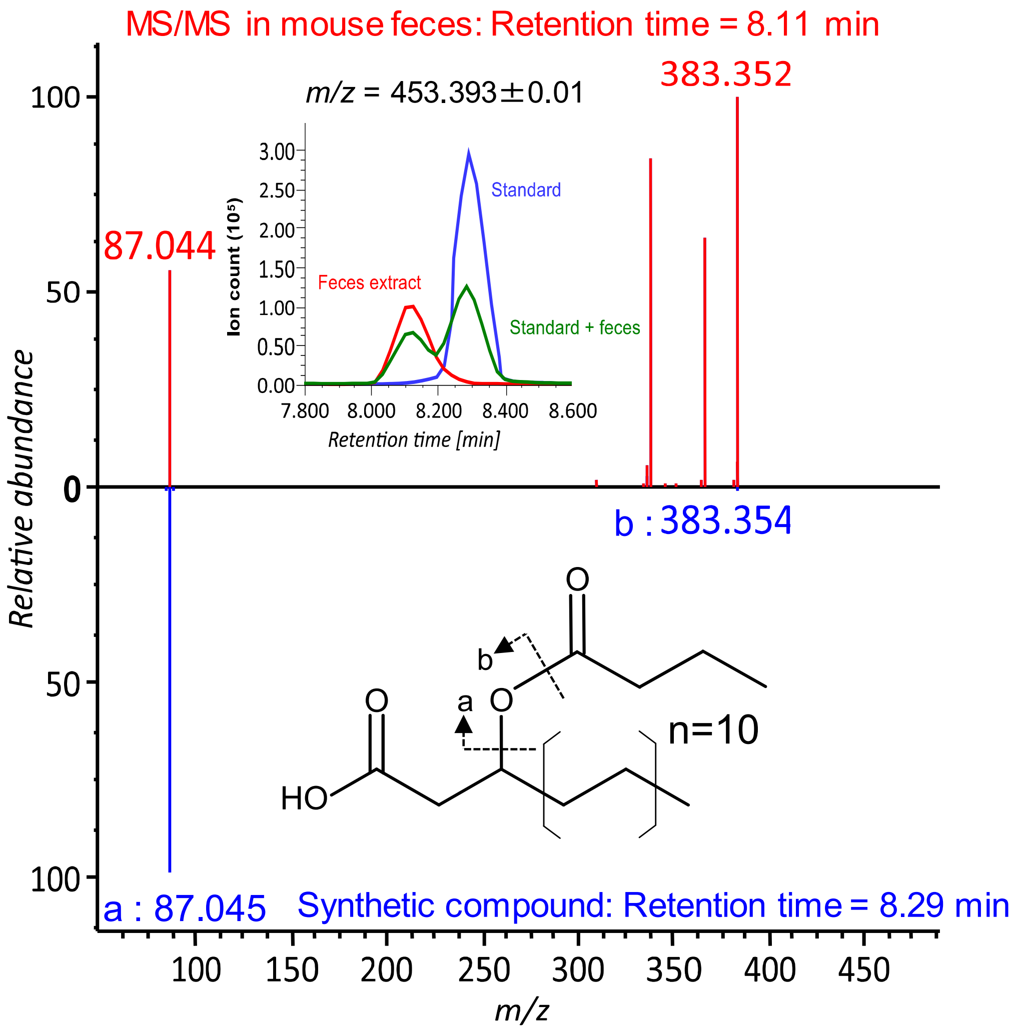

Figure S2. Comparison of FAHFA in mouse feces and synthetic butyric acid esters of β-hydroxy fatty acid, Related to Figure 3

MS/MS spectra in the feces (red) and synthetic standard of butyric acid esters of β-hydroxy fatty acid (blue) were presented. The chemical structure represents the fragmentation of butyric acid esters of β-hydroxy fatty acid. The chromatograms of the fecal extract (red), synthetic standard of butyric acid esters of β-hydroxy fatty acid (blue), and their mixture (green) were also described.

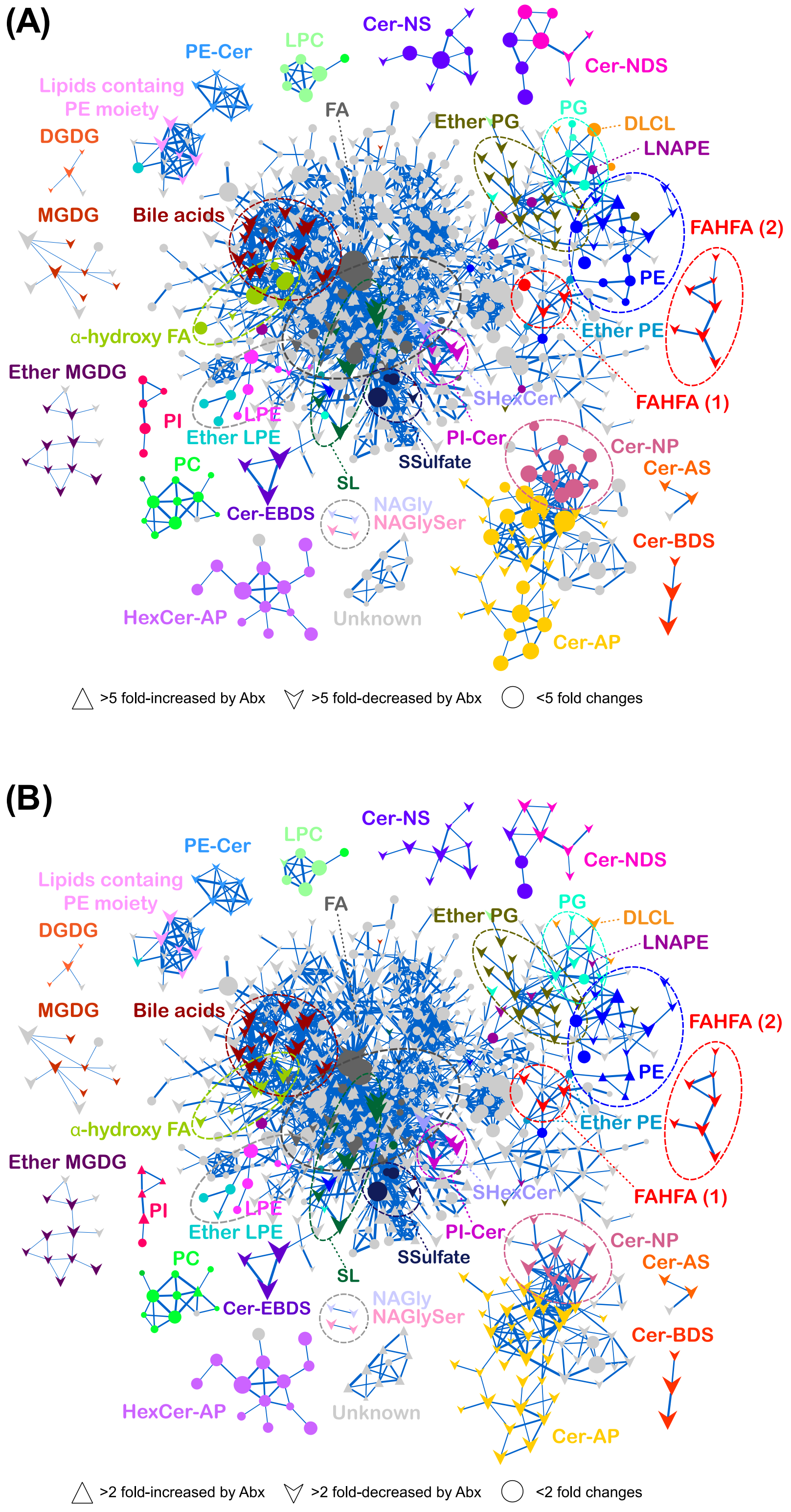

Figure S3. Feature-based molecular spectrum networking of fecal lipidome, Related to Figure 2

The nodes of circle, and up- and down-arrows represent lipid ions with less than 2, and 5-fold changes, and more than 2, and 5-fold increase and decrease, respectively, in the Abx treatment group, when compared to the control group.

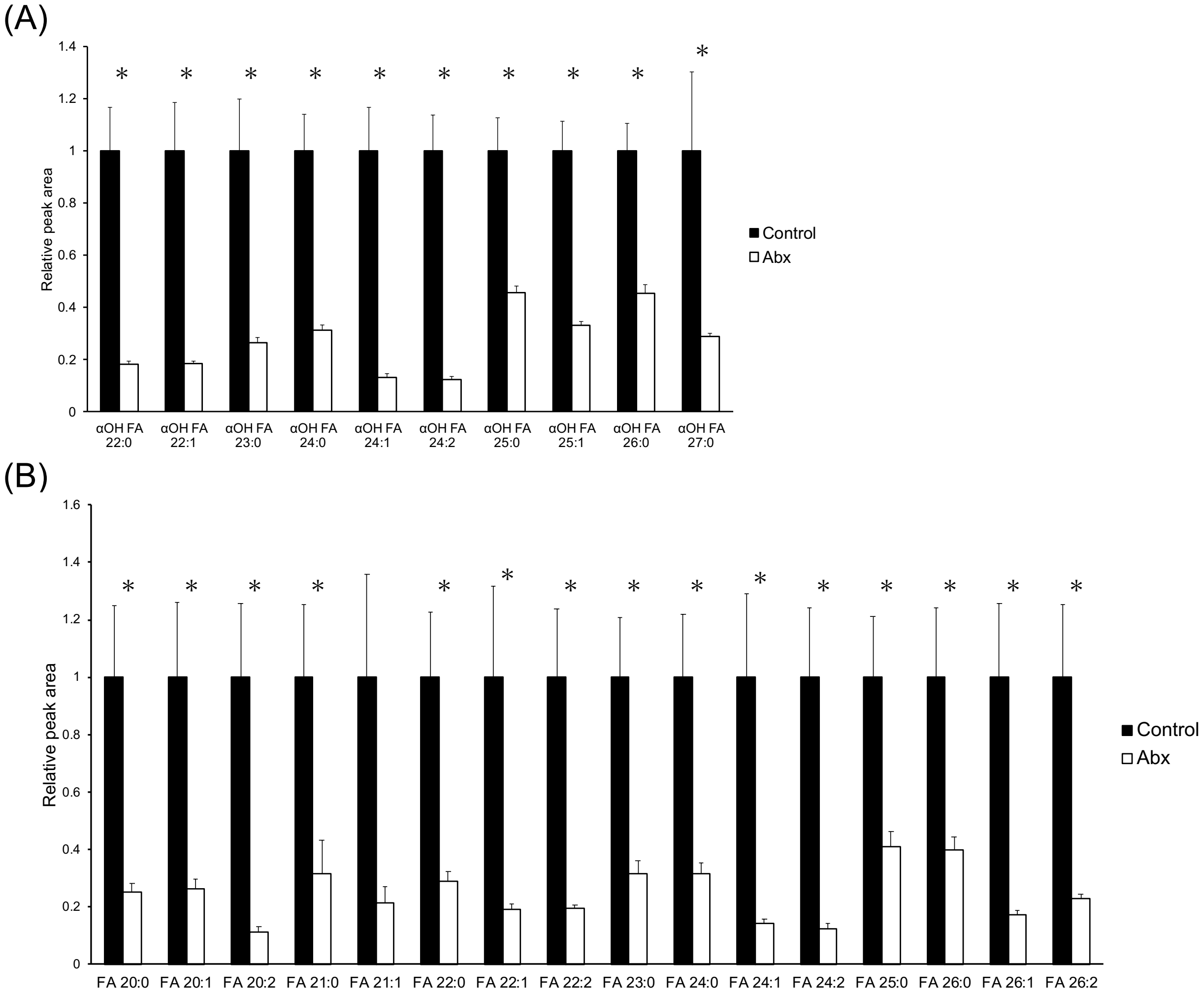

Figure S4. Relative amounts of α-hydroxy fatty acids and VLCFAs in mice feces, Related to Figure 2

Relative peak areas of (A) α-hydroxy fatty acids and (B) VLCFAs in control and Abx-treated mouse feces. Data are means ± SEM (n=5). Data were analyzed by Student's t-test (∗ : $p < 0.01$).

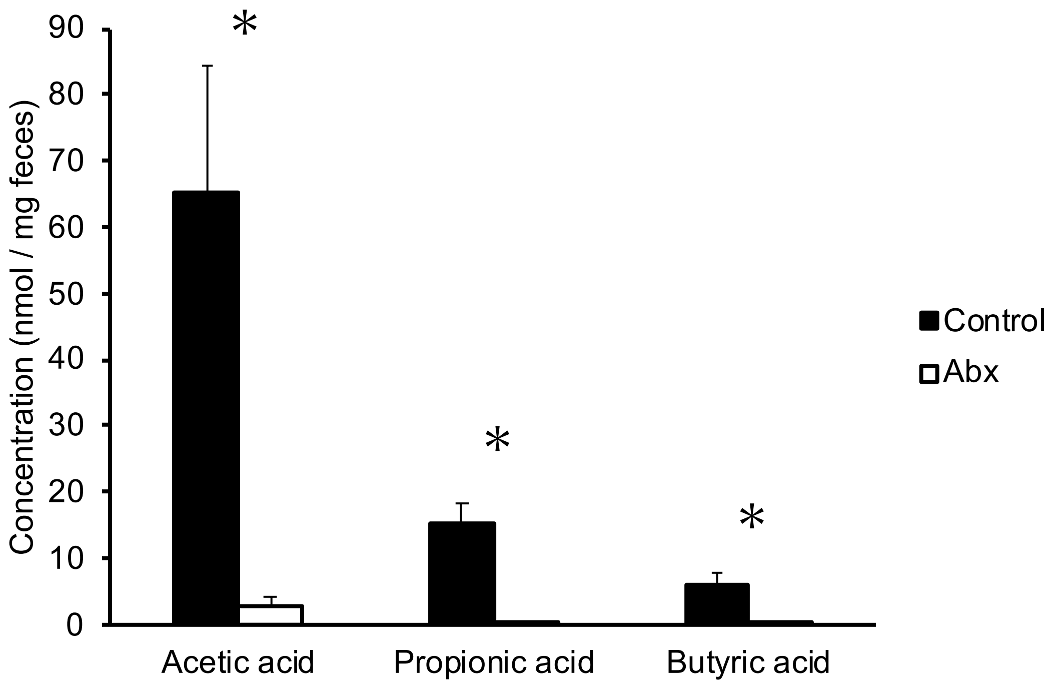

Figure S5. Concentrations of SCFAs in control and Abx-treated mouse feces, Related to Figure 2

Concentrations of acetic acid, propionic acid, and butyric acid in control and Abx-treated mouse feces. Data are mean ± SEM (n=5). Data were analyzed by Student's t-test (* : $p < 0.01$).

Supplemental data

Data S1. 16S rRNA sequence data, Related to Figure 1

Data S2. Targeted and untargeted lipidomics data, Related to Figure 2

Data S3. Nomenclature of lipids, Related to Figure 2

Transparent Methods

Mice and antibiotics

Male wild-type C57BL/6 from CLEA Japan, Inc. (Tokyo, Japan) were maintained in a specific pathogen-free environment. Ampicillin (1 g/L), vancomycin (0.5 g/L), neomycin (1 g/L), and metronidazole (1 g/L) in drinking water was administrated for two weeks from six weeks of age. Five mice were used in each experimental group. All antibiotics were purchased from Wako Pure Chemicals Corporation (Osaka, Japan). All animal experiments were approved by the Animal Care and Use Committee of Riken Yokohama Institute (No. 2019-015(2)).

Lipidomics

Collected mice feces were frozen in liquid nitrogen and homogenized by shaking with metal corn using multi beads shocker (YASUI KIKAI, Osaka, Japan) for 15 sec at 2500 rpm in a plastic vial. To the homogenized sample, 100 μL methanol per 10 mg-feces was added and homogenized in the identical condition. The homogenate was incubated overnight at -30℃. Whole lipids were extracted by single-phase extraction (Tsugawa et al., 2020). Briefly, 200 μL of fecal suspension in methanol was mixed with 100 μL chloroform and incubated for 2 h at room temperature. Subsequently, 20 μL of water was added, and the samples were incubated for 10 minutes. After extraction, samples were centrifuged at 2000×*g* for 10 min, and the supernatants were collected. Untargeted lipidomics was performed by using an ACQUITY UPLC system (Waters, Milford, MA) coupled with a QTOF-MS (TripleTOF 5600^{+} or TripleTOF 6600; Sciex, Framingham, MA) (Tsugawa et al., 2020). Lipids were separated on an Acquity UPLC Peptide BEH C18 column (50 × 2.1 mm; 1.7 μm) (Waters, Milford, MA, USA). The column was maintained at 45°C at a flow-rate of 0.3 mL/min. The mobile phases consisted of (A) 1:1:3 (v/v/v) acetonitrile:methanol:water with ammonium acetate (5 mM) and 10 nM EDTA and (B) 100% isopropanol with ammonium formate (5 mM) and 10 nM EDTA. The separation was conducted under the following gradient: 0 min 0% (B); 1 min 0% (B); 5 min 40% (B); 7.5 min 64% (B); 12 min 64% (B); 12.5 min 82.5% (B); 19 min 85% (B); 20 min 95% (B); 20.1 min 0% (B); and 25 min 0% (B). The sample temperature was maintained at 4°C. MS analysis was performed at the high sensitivity mode in MS2. Data-dependent MS/MS acquisition (DDA) was used. The parameters were MS1 and MS2 mass ranges, *m/z* 70–2000; MS1 accumulation time, 250 ms; MS2 accumulation time, 100 ms; collision energy, 40 eV; collision energy spread, 15 eV; cycle time, 1300 ms; curtain gas, 30; ion source gas 1, 50(-); ion source gas 2, 50(-); temperature, 300°C(-); ion spray voltage floating, –4.5 kV; and declustering potential, 80 V. The other DDA parameters were dependent product ion scan number, 10; intensity threshold, 100 cps; exclusion time of precursor ion, 0s; mass tolerance, 20 ppm; ignore peaks, within *m/z* 200; and dynamic background subtraction, True. The mass calibration was automatically performed using an APCI positive/negative calibration solution via a calibration delivery system. MS-DIAL bootstrap version 3.96 was used (the lipid subclasses characterized in this study can also be annotated by the publicly available versions (4.10 or later) (Tsugawa et al., 2020). The following parameters were set: (data collection) RT begin, 0.5 min; retention time end, 18 min; mass range begin, 0 Da; mass range end, 2000 Da; MS1 tolerance, 0.01 Da; MS2 tolerance, 0.025 Da; (peak detection) minimum peak height, 500 amplitude; mass slice width, 0.1 Da; smoothing method, linear weighted moving average; smoothing level, 3 scan; minimum peak width, 5 scan; exclusion mass list, none; (identification) retention time tolerance, 2 min; MS1 accurate mass tolerance, 0.01 Da; MS2 accurate mass tolerance, 0.05 Da; identification score cut off, 80%; (alignment) retention time tolerance

0.05 min; MS1 tolerance, 0.015 Da. Default values were used for other parameters. Oxidized fatty acids, including linoleic acid metabolites, were extracted by Sep-Pak C18 cartridge (Waters) and analyzed by LC-QqQMS according to previous reports (Kishino et al., 2013, Naoe et al., 2019).

Feature-based molecular MS/MS spectrum networking

The procedure was performed for the alignment peak properties of MS-DIAL having MS/MS spectral information, where the retention time, precursor *m/z*, and MS/MS spectrum of known/unknown lipids are utilized. The node (detected peak feature) was connected by the similarity of MS/MS spectra, based on a previously reported method (Tsugawa et al., 2019). As preprocessing, the MS/MS spectrum was segmented by 100 Da, and the top five most intense peaks were retained where the intensity was transformed by square root, and the base peak was normalized to 1000. Given that two spectra are compared, the spectrum of the larger precursor ion (spectrum A) was templated, and spectrum B was fitted to spectrum A. The peak was defined as a 'match' when the product ion or neutral loss of spectrum A and B was within 0.025 Da. The fragment ions which are not matched to any of the template spectra are defined as 'unmatched'. The Bonanza score (Falkner et al., 2008) using the matched and unmatched fragment (transformed) intensities was utilized to measure MS/MS similarity, and the score cut off was set to 85% in this study. The node and edge tables were imported to Cytoscape 4.0 program (https://cytoscape.org/). According to the latest literature of GNPS environment (FBMN; https://www.nature.com/articles/s41592-020-0933-6) and the paper reporting the detailed algorithm of the GNPS molecular network (https://www.pnas.org/content/109/26/E1743), the GNPS program uses cosine score instead of Bonanza score, and the threshold of minimum matched peaks is used to define the MS/MS network (default: >5 matched peaks). Although the result of the MS-DIAL molecular network should be different from that of the GNPS environment, both programs contribute to the increase of the annotation rate in metabolomics and lipidomics studies.

SCFAs analysis

SCFAs were simultaneously extracted and derivatized by using pentafluorobenzyl bromide alkylation reagent (Thermo Fischer Scientific) from 5.0 mg feces homogenate in methanol or 20 μL of plasma mixed with deuterium-labeled internal standard (1.0 mM of acetate-d3, 1.0 mM of propionate-d5, and 1.0 mM of butyrate-d7 (Cambridge isotope laboratories)) following the manufacturer's protocol. The derivatized SCFAs were analyzed by GCMS TQ8030 (Shimadzu, Kyoto, Japan) equipped with a BPX-5 column (30 m, 0.25 mmID, 0.25 μm, SGE Analytical Science, Melbourne, Australia). The fragment ions of *m/z* = 240, 243, 254, 259, 268, and 275 for the derivatized acetate, acetate-d3, propionate, propionate-d5, butyrate, and butyrate-d7, respectively, were measured in selected ion monitoring mode. The analysis conditions were as follows: injection volume, 1μL; injection mode, split (split ratio, 1:10), flow rate, 1.0 ml/min; carrier gas, helium; oven temperature gradient, 60°C for 2 min, increased at a rate of 20°C/min to 240°C and maintained for 3min; interface temperature, 250°C; and ionization, electron ionization (70 eV).

Synthesis of acyl alpha and beta hydroxyl fatty acids

Acyl alpha and beta hydroxyl fatty acids were synthesized by adding 2 μL of butyric chloride (19.2 mmol) (Sigma

Aldrich, Tokyo, Japan) to 300 µL of THF containing 5 mM of cerebronic acid or 3-hydroxytetracosanoic acid (1.5 mmol) (MATREYA LCC, PA, USA) and 1% v/v trimethylamine (21.6 mmol) (Tokyo Chemicals Industry, Tokyo, Japan). The solution was stirred overnight at 37℃, then 100 µL of water was added to hydrolyze excess butyric chloride. The synthesized product was purified using high-pressure liquid chromatography (Prominence, Shimadzu, Kyoto, Japan) equipped with a UV detector and Waters XBridge C18 column (100 mm × 4.6 mm inner diameter, 5 µm particle size, Waters). The flow rate was set at 1.0 ml/min. The other conditions were identical to that of LC-QTOF-MS analysis.

Extraction, sequencing, and data processing of the bacterial 16S rRNA gene

Microbial DNA in mice feces was extracted by using QIAamp fast DNA stool mini kit (QIAGEN, Hilden, Germany) following the manufacturer's protocol. The frozen feces (~50 mg) were homogenized by beads beating for 10 min at 1500 rpm using zirconia beads with 0.1 mm and 3 mm diameter in 500 µL of inhibitEX buffer. The preparation of amplicon and sequencing was performed following the 16S Metagenomic Sequencing Library Preparation protocol on MiSeq platform (Illumina, CA, USA). The variable V3 – V4 regions of the 16S rRNA gene were amplified by polymerase chain reaction using universal primers. The pair-end reads were joined with the ea-utils program (https://github.com/ExpressionAnalysis/ea-utils). The joined reads with an average quality value < 25 and lacking the primer sequences at both ends were then excluded. The filtered and trimmed 3,000 reads were randomly selected, and the selected reads were sorted according to the quality of the reads, and then clustered into OTUs using a 97% pairwise identity cutoff with the USEARCH program (v.10.0.240) (Edgar et al., 2010). Representative sequences of the generated OTUs were searched for their similarity using the GLSEARCH program (v. 36.3.8e) against publicly available 16S rRNA gene databases described previously (Takayasu et al., 2017). For assignment of phylum, order, family, genus and species levels, a sequence similarity threshold of 70%, 80%, 90%, 95%, and 97% was applied, respectively.

Statistical analysis

Principle component analysis was performed by R (version 3.6.1) using the "rgl" package (version 0.100.50). Spearman's rank correlation was calculated with the SciPy statistical package (version 1.1.0) on python (version 3.6.1.0). Hierarchical clustering was performed using the seaborn package (version 0.9.0). PCoA was performed as described previously (Iwasawa et al., 2018).

Supplemental reference

Edgar, R.C. (2010). Search and clustering orders of magnitude faster than BLAST. Bioinformatics *26*, 2460–2461.

Falkner, J.A., Falkner, J.W., Yocum, A.K., and Andrews, P.C. (2008). A spectral clustering approach to MS/MS identification of post-translational modifications. J. Proteome Res. *7*, 4614–4622.

Iwasawa, K., Suda, W., Tsunoda, T., Oikawa-Kawamoto, M., Umetsu, S., Takayasu, L., Inui, A., Fujisawa, T., Morita, H., Sogo, T., et al. (2018). Dysbiosis of the salivary microbiota in pediatric-onset primary sclerosing cholangitis and its potential as a biomarker. Sci. Rep. *8*, 1–10.

Kishino, S., Takeuchi, M., Park, S.B., Hirata, A., Kitamura, N., Kunisawa, J., Kiyono, H., Iwamoto, R., Isobe, Y., Arita, M., et al. (2013). Polyunsaturated fatty acid saturation by gut lactic acid bacteria affecting host lipid composition. Proc. Natl. Acad. Sci. U. S. A. *110*, 17808–17813.

Naoe, S., Tsugawa, H., Takahashi, M., Ikeda, K., and Arita, M. (2019). Characterization of lipid profiles after dietary intake of polyunsaturated fatty acids using integrated untargeted and targeted lipidomics. Metabolites *9*.

Tsugawa, H., Nakabayashi, R., Mori, T., Yamada, Y., Takahashi, M., Rai, A., Sugiyama, R., Yamamoto, H., Nakaya, T., Yamazaki, M., et al. (2019). A cheminformatics approach to characterize metabolomes in stable-isotope-labeled organisms. Nat. Methods *16*, 295–298.

Tsugawa, H., Ikeda, K., Takahashi, M., Satoh, A., Mori, Y., Uchino, H., Okahashi, N., Yamada, Y., Tada, I., Bonini, P., Higashi, Y., Okazaki, Y., Zhou, Z., Zhu, Z. J., Koelmel, J., Cajka, T., Fiehn, O., Saito, K., Arita, M., Arita, M., (2020). A lipidome atlas in MS-DIAL 4. Nat. Biotechnol. in press

Takayasu, L., Suda, W., Takanashi, K., Iioka, E., Kurokawa, R., Shindo, C., Hattori, Y., Yamashita, N., Nishijima, S., Oshima, K., et al. (2017). Circadian oscillations of microbial and functional composition in the human salivary microbiome. DNA Res. *24*, 261–270.
